# Dilatometric and Microstructural Study of Martensite Tempering in 4% Mn Steel

**DOI:** 10.3390/ma13194442

**Published:** 2020-10-07

**Authors:** Adam Grajcar, Mateusz Morawiec, Jose Antonio Jimenez, Carlos Garcia-Mateo

**Affiliations:** 1 Department of Engineering Materials and Biomaterials, Silesian University of Technology, 44-100 Gliwice, Poland; mateusz.morawiec@polsl.pl; 2 Department of Physical Metallurgy, National Center for Metallurgical Research, 28040 Madrid, Spain; jimenez@cenim.csic.es (J.A.J.); cgm@cenim.csic.es (C.G.-M.)

**Keywords:** quenching, tempering, medium-Mn steel, martensitic transformation, dilatometry, cementite precipitation

## Abstract

This paper presents the results of martensite tempering resistance in 4% Mn steel. The material was quenched and tempered at 350 °C for 15, 30, and 60 min. The analysis of the quenching and tempering was carried out using dilatometric and microstructural approaches. The phase composition was assessed using X-ray diffraction. The M_s_ temperature and tempering progress were simulated using JMatPro software. The dilatometric analysis revealed a small decrease in the relative change in length (RCL) during tempering. This decrease was connected to the precipitation kinetics of cementite within the martensite laths. The microstructure investigation using a scanning electron microscope showed a very small amount of carbides, even for the longest tempering time. This showed the high tempering resistance of the martensite in medium-Mn steels. The hardness results showed an insignificant decrease in the hardness depending on the tempering time, which confirmed the high tempering resistance of martensite.

## 1. Introduction

Medium manganese steel is a grade of steel that uses Mn as an important alloying element for the stabilization of retained austenite at room temperature. This is because manganese is an austenite stabilizer [1]. This microstructural feature is used in different types of heat treatments, which usually result in ferrite, bainite, or martensite formation (as a matrix), with some fraction of retained austenite. The typical heat treatment for this grade of steel is the intercritical annealing of cold-rolled steel [2,3]. Here, the formation of a mixture of α + γ phases from low-carbon martensite takes place. The formation of the ferrite leads to the diffusion of carbon and manganese (for sufficiently long times) into the austenite. This element increases its thermal stability, which favors higher amounts of the γ phase at room temperature. Another possible heat treatment is austempering for the bainite formation after austenitization [4,5]. The formation of bainite stimulates a higher carbon content of the remaining austenite. Recently, some research has focused on the use of a two- or three-step heat treatment using intercritical annealing or isothermal holding [6,7,8]. Very complex microstructures can be obtained in this way, leading to better mechanical properties. All of these treatments use the rejection phenomenon of carbon and manganese from the alpha phases.

Recently, a very popular heat treatment is quenching and partitioning (QP), where first, some fraction of martensite is formed during partial quenching. Then, the material is heated above the martensite start temperature (M_s_), resulting in martensite tempering and carbon partitioning [9]. Depending on the partitioning temperature (from 200 to 500 °C), different phases, such as bainite or pearlite, can be formed [10]. The higher the temperature of the isothermal holding after the partial quenching, the larger the grain size of the matrix. However, when the temperature is close to M_s_, the diffusion needs more time for carbon partitioning into the austenite.

The tempering behavior is well known for conventional low-alloyed or high-alloyed steels [11,12]. However, there are no systematic studies on the tempering resistance in advanced medium-Mn steels. The QP process uses martensite tempering as a source of carbon and manganese (at higher partitioning temperatures) for austenite stabilization. So far, there is not enough knowledge regarding how the tempering time affects the martensite resistance in steels containing above 3% Mn. Therefore, this work was focused on the analysis of martensite tempering near the M_s_ temperature for different tempering durations.

## 2. Material and Experiments

### 2.1. Material

The chemistry of the experimental laboratory-produced medium-Mn steel is shown in Table 1. The steel belongs to the newly developed third-generation of AHSS (advanced high strength steel) [1,3]. The chemical composition was analyzed using glow discharge optical emission spectrometry (GDOES). This steel was designed for the bainite transformation heat treatment to obtain a microstructure composed of a bainitic matrix with some fraction of retained austenite. The manganese content of 3.6% was added as the γ-phase stabilizer for the retained austenite stabilization [1]. A high Al content was introduced to reduce the negative effect of Si on the sheet wettability by liquid zinc during the hot-dip galvanizing [13]. These elements were used to prevent carbide precipitation during the bainite transformation. The small Nb content was added to limit the prior austenite grain growth during austenitization [14].

### 2.2. Experimental Details

The main purpose of this work was to analyze the martensite tempering resistance during a heat treatment near the M_s_ temperature. The work consisted of two parts, including quenching and tempering of the investigated steel. The dilatometric study of the M_s_ temperature and the tempering kinetics were carried out using a high-resolution BAHR Dilatometer DIL805A/D (TA Instruments, Wetzlar, Germany) with induction heating of the samples in a vacuum. The critical temperatures were determined based on the ASTM A1033-04 standard [15]. The samples were 4 mm in diameter and 10 mm in length and were machined for the dilatometric analysis. The cooling was conducted using helium.

**Figure 1:**
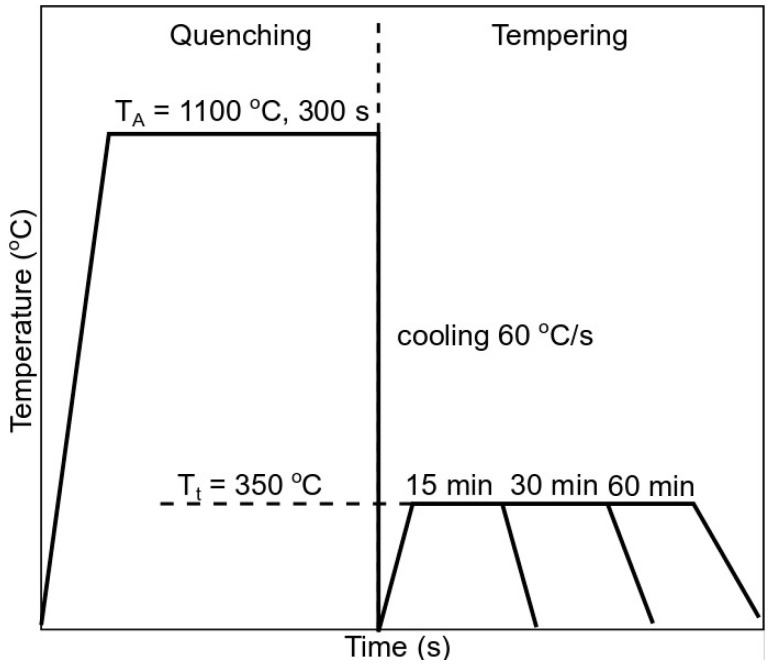
A schematic of the heat treatment.

The steel was austenitized at 1100 °C for 300 s and cooled down at a rate of 60 °C/s to room temperature to obtain a fully martensitic microstructure. Then, the dilatometric samples were tempered at 350 °C for 15, 30, and 60 min, as presented in Figure 1.

After this, the microstructure analysis using a light microscope and scanning electron microscope, as well as X-ray diffraction, were performed. For metallographic purposes, the samples were cut in half, ground, and polished using 3 and 1 µm diamond pastes, after which, etching in 2% Nital (the solution of 2% nitric acid with the alcohol) was conducted. The hardness measurements were carried out using the Vickers method with a load of 100 N for 15 s. In this way, the softening behavior of the martensite during tempering was assessed. JMatPro (Version 11.2, Sente Software, Guildford, UK) simulations during quenching and tempering steps were carried out for further analysis of the microstructure changes [16]. The database version 11.2 and the general steel module were used for the simulations.

X-ray diffraction (XRD) measurements were carried out using a Bruker AXS D8 diffractometer (Bruker AXS, GmbH, Karlsruhe, Germany) equipped with a Co X-ray tube working at 40 kV and 30 mA, as well as a LynxEye Linear Position Sensitive Detector (Bruker AXS, GmbH, Karlsruhe, Germany), which allowed for data acquisition up to 450 times faster than a conventional point detector system. We used Co radiation to avoid the strong fluorescent radiation that iron emits when copper radiation is used, which causes a significant decrease in the intensity of the diffraction peaks and the low-intensity lines to be submerged into the contribution of fluorescence to the background. In the equipment configuration used, a parallel beam of monochromatic X-ray radiation was produced by introducing a parabolically bent graded multilayer Göbel mirror in the path of the primary beam. Conventional θ–2θ scans were carried out over a 2θ range of 45–135° with a step size of 0.01° on samples prepared following the metallographic procedures described before. The experimental setup used allowed for obtaining high-resolution diffraction patterns that showed over 15,000 counts for the most intense lines. Therefore, the limit of detection (LoD) of a phase could be set at 0.3 wt% when its strongest peaks did not overlap with diffraction peaks of other phases. Furthermore, the use of the Rietveld method to evaluate these patterns allowed for performing quantitative analyses with relative errors lower than 20% in stable fits with good precision only for contents higher than 1.0 wt%. In this work, the 4.2 version of the Rietveld analysis program TOPAS (Bruker AXS, GmbH, Karlsruhe, Germany,) was used for the XRD data refinement.

## 3. Results and Discussion

### 3.1. Quenching Behavior

**Figure 2:**
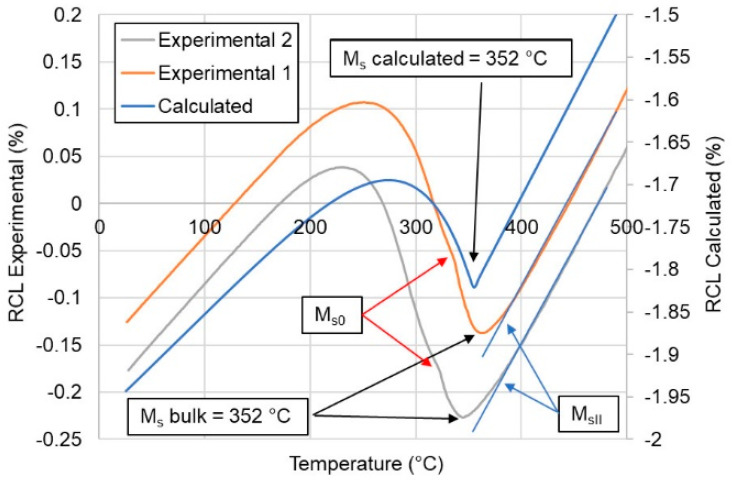
Analysis of the martensitic transformation according to the JMatPro simulation and the dilatometric study. RCL: relative change in length.

The first step of the experiment was the quenching of the medium manganese steel. The JMatPro simulation was carried out to determine the martensite start temperature (M_s_) of the steel. Then, the dilatometric analysis of the quenching process was conducted. Two samples were quenched to ensure the reproducibility of the experiment. According to the computational and dilatometric results (Figure 2), the calculated M_s_ temperature of the steel was 352 °C. The dilatometric curves showed the same bulk M_s_ temperature for the analyzed steel. However, there were some differences in the curves. First, it can be seen that the martensite transformation started at a slightly higher temperature of 390 °C (M_sII_—represents a temperature at which small amount of lower stability austenite transforms from γ to α’ just before the bulk transformation). Then, at 352 °C, the bulk martensitic transformation occurred. At the same time, a small increase in the relative change in length (RCL) in both samples occurred after the bulk transformation (M_s0_—represents a temperature just after the bulk transformation, at which another small amount of highly stable austenite is transformed into martensite). This means that at around 335 °C, another martensite transformation occurred. This result means that the austenite undergoing the martensitic transformation had a different chemical composition in various microregions. We confirmed this effect in the microstructural investigations (banding appeared). This behavior resulted in a three-step transformation during quenching. First, a small amount of austenite with the lowest thermal stability underwent a transformation; then, the bulk transformation of the austenite took place, and finally, the austenite with the highest thermal stability was transformed. This effect could be the result of local variations in the chemical composition due to the segregation of Mn during solidification. The hot-working enhanced the manganese redistribution in segregation bands. This difference in the local manganese content also influenced the local M_s_ temperature. According to Hidalgo et al. [17], Mn-rich regions decrease the local martensite start temperature compared to Mn-poor regions. Therefore, Mn can influence the M_s_ temperature of the steel similarly to that presented for carbon in Figure 2. Moreover, manganese reduces the C activity and its diffusion rate [18], which affects the martensite formation during quenching.

Another factor influencing the M_s_ temperature is related to the partial dissolution of niobium carbides. According to Garcia de Andres et al. [19], the occurrence of the steps during a martensite transformation could be the result of the partial dissolution of carbides during austenitization. In the analyzed case, this could be the result of the partial dissolution of niobium carbides during the austenitization step at 1100 °C. The austenite where the carbides were dissolving should have higher carbon concentrations compared to the areas where the carbides did not dissolve. It seems that this effect was smaller compared to the one related to the Mn segregation.

**Figure 3:**
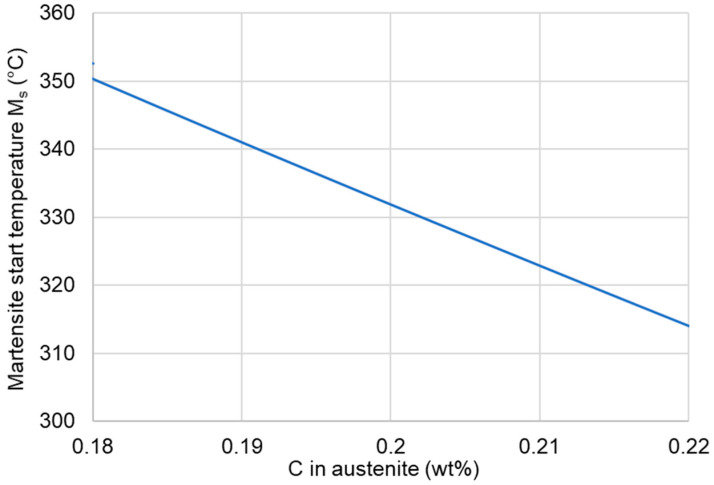
Martensite start temperature calculated using JMatPro.

According to the calculation results shown in Figure 3, the M_s0_ temperature of 335 °C indicates that the austenite should have a carbon content of ~0.2 wt%. At the same time, the small transformation of austenite at the beginning means that it had a lower carbon content compared to the bulk composition. However, after comparing the C and Mn effects on the austenite decomposition, it seems that the manganese segregation can be a critical issue in terms of the M_s_ temperature in medium-Mn steels [20,21].

### 3.2. Tempering Behavior

**Figure 4:**
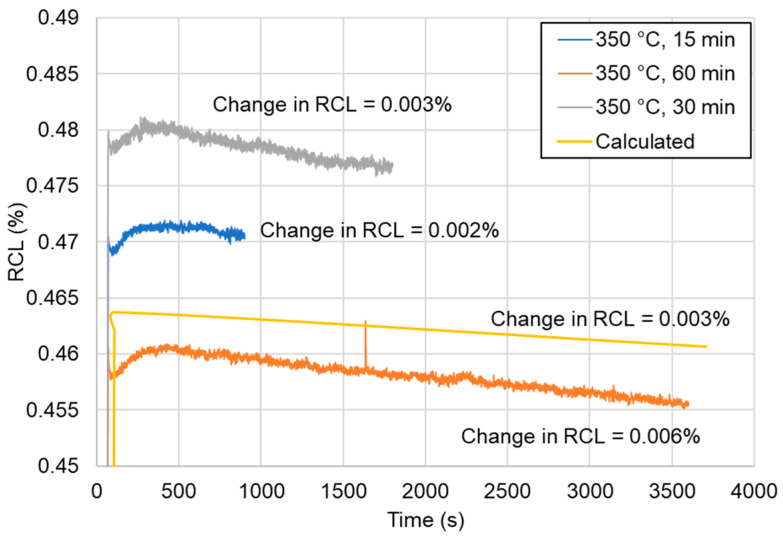
Tempering progress according to the dilatometric results and the JMatPro simulation.

The next step of the study was to analyze the tempering process of the steel after quenching. The tempering was carried out at 350 °C for 15, 30, and 60 min to investigate whether it is possible for martensite to undergo tempering at this relatively low temperature. According to the dilatometry results presented in Figure 4, the change of the RCL was negligible for all selected times. This change was calculated via the subtraction of the lowest value of the RCL from the higher value for a selected time. The drop in RCL was correlated with the precipitation of carbides during tempering. The obtained experimental results were in good agreement with the simulation data performed using JMatPro. The simulation was performed with the martensitic microstructure being heated up at a rate of 3 °C/s to 350 °C and tempered for 60 min. The types of carbides taken into account in the simulations for the Fe alloys model were M_6_C, M_7_C_3_, and M_23_C_6_. The results of the simulation presented a neglectable shrinkage during tempering over 60 min. One can see that with a longer tempering time, a higher drop in the RCL took place. However, the small level of this decrease indicated a lack (or a very limited extent) of any precipitation of carbides. The carbides could be formed locally but the amount of them should be very small. Therefore, they could not influence the properties of the martensite in any way. The reason for this can be related to the Mn effect on the carbon diffusion. As mentioned above, Mn decreases the carbon diffusion rate in steel [18]. This means that the selected time (60 min) was too short for carbon to have enough time to form carbides. This was in good agreement with the dilatometric results.

**Figure 5:**
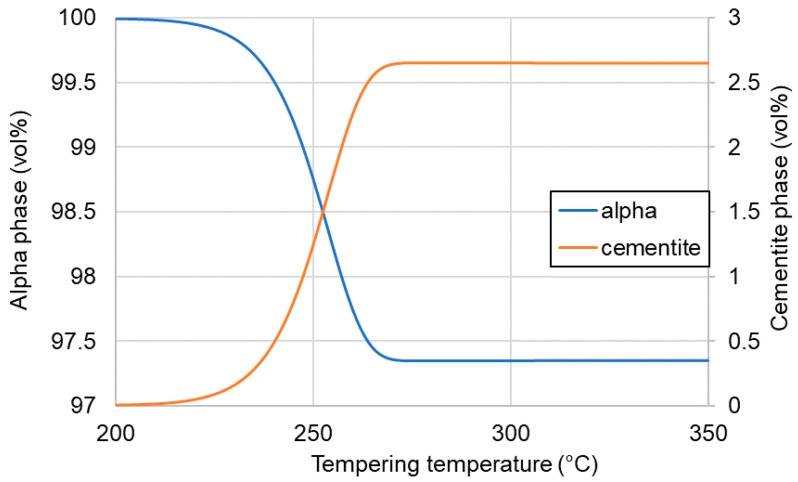
Cementite precipitation level at different tempering temperatures.

To estimate the possible amount of carbides at a temperature of 350 °C, calculations were made and the obtained results are presented in Figure 5. The maximum amount of carbides (cementite according to the simulations) that could be precipitated during 60 min of tempering was around 2.5%. This value was constant from the tempering temperature of 275 °C and did not increase up to 350 °C. This means that the contraction of the dilatometry curve could be the result of the formation of ~2.5% carbides during tempering.

### 3.3. Microstructure

**Figure 6:**
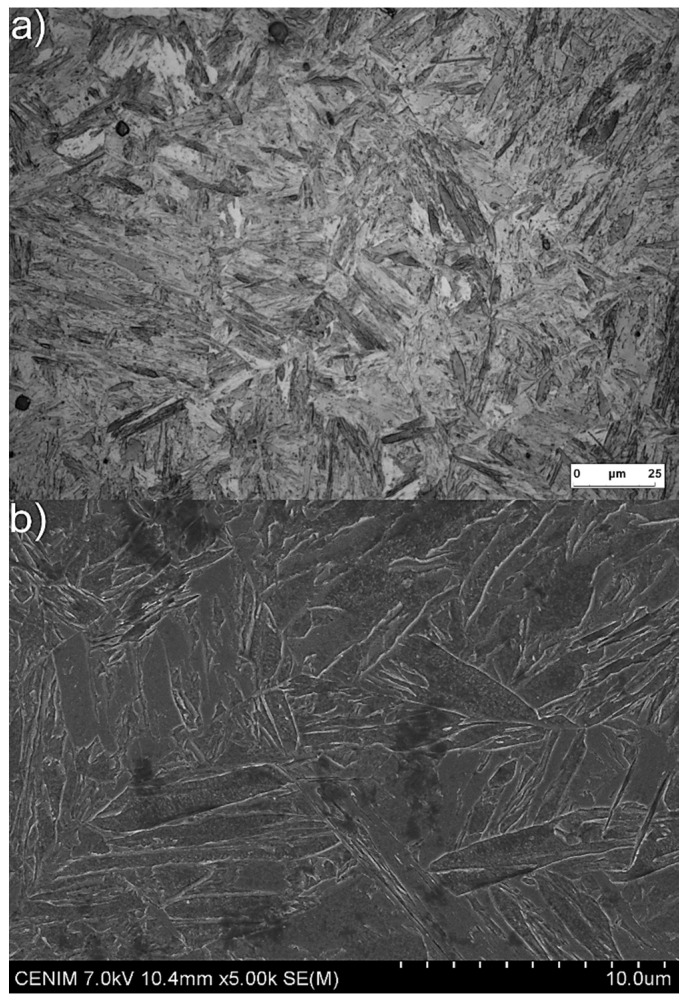
Martensitic microstructures of steel after quenching: (**a**) light microscopy (LM) and (**b**) scanning electron microscopy (SEM).

After quenching, the microstructure of the medium-Mn steel was fully martensitic (Figure 6). No retained austenite or any other phases were visible in the microstructure. This was the result of the high hardenability effect of manganese [22]. Note that the austenitization was full despite the high concentration of Al in the steel, which increased the A_c3_ temperature.

**Figure 7:**
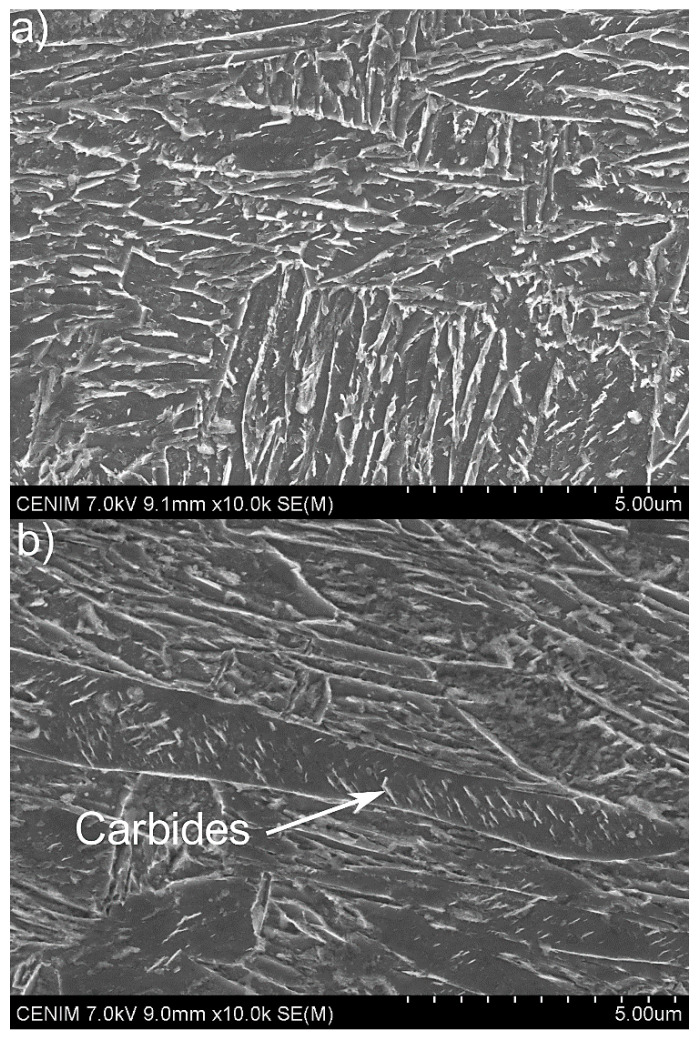
The SEM microstructures of the steel after tempering for (**a**) 15 and (**b**) 60 min.

After tempering (Figure 7), the microstructure did not change much. The microstructure after 15 min (Figure 7a) did not show any distinct changes in the morphology of the martensite and no cementite precipitates were visible. Looking at the martensite after 60 min of tempering, some small changes in its morphology were visible. Moreover, some precipitates inside the martensite laths could be seen. According to Bhadeshia [23], the carbides with a plate-like shape that are presented in Figure 7b (indicated by a white arrow) come from the tempering process of martensite. This means that some tempering effect took place. However, its effect was not too strong. Thus, the martensite in the analyzed medium-Mn steel had a high tempering resistance at 350 °C. Even 1 h tempering was not enough to significantly start the precipitation process.

### 3.4. X-ray Diffraction

**Figure 8:**
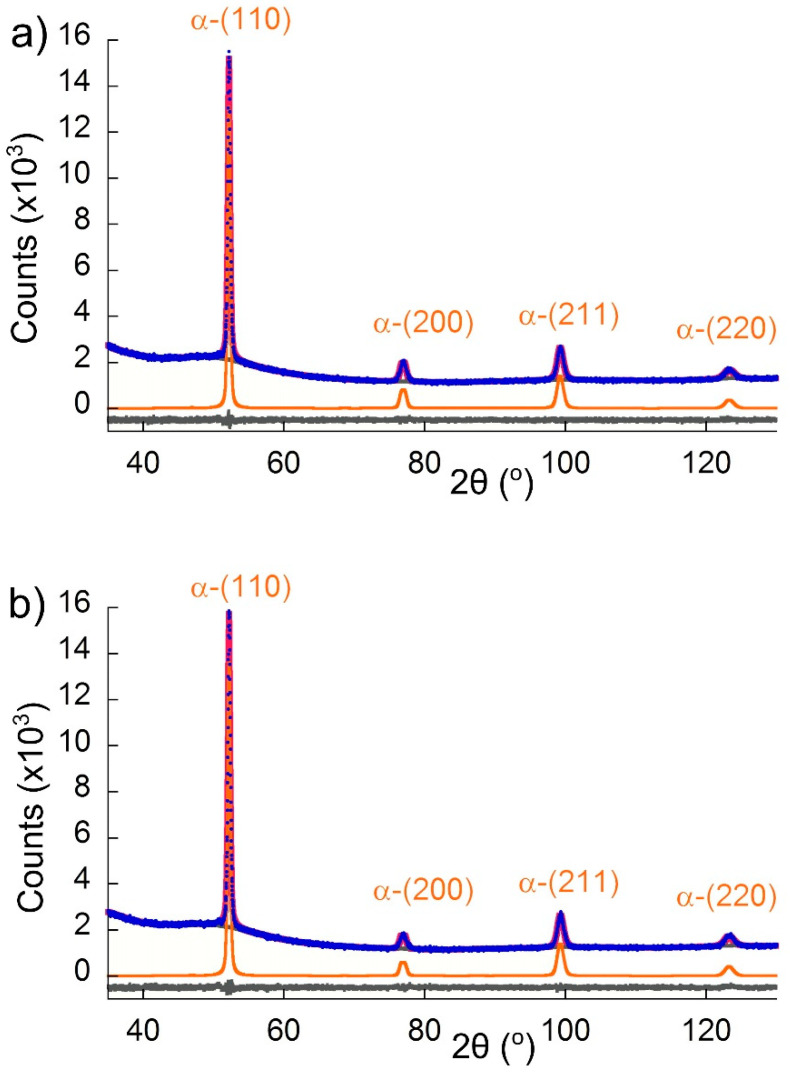
X-ray diffraction patterns of the samples after tempering for: (**a**) 15 and (**b**) 60 min (blue line—experimental pattern, red line—calculated pattern, gray line—differential curve).

To check the amount and type of carbides in the tempered microstructure, X-ray diffraction was carried out. In Figure 8, the observed (blue points) XRD patterns were compared with the calculated (red solid line) profiles obtained after a Rietveld refinement. The differential curve is shown as a gray line and the background removed phase (α phase) is shown in orange. According to the result presented in this figure, no carbides were detected after 15 (Figure 8a) or 60 min (Figure 8b) of tempering. This means that either the carbide precipitation did not occur or that their amount and size was below the detection limit of the technique. The latter seems to be the case, as the JMatPro simulations and SEM investigations in Figure 7 indicate some precipitation took place. The XRD patterns in Figure 8 also show that no peaks of retained austenite were present, but only the following α phase peaks were: (110), (002), (211), and (022). Moreover, if some retained austenite was present before quenching, the γ to α transformation should be detected via dilatometry, and as Figure 4 indicates, this was not the case. In brief, no retained austenite was present in the microstructure after either of the tempering conditions that were analyzed.

### 3.5. Hardness

**Figure 9:**
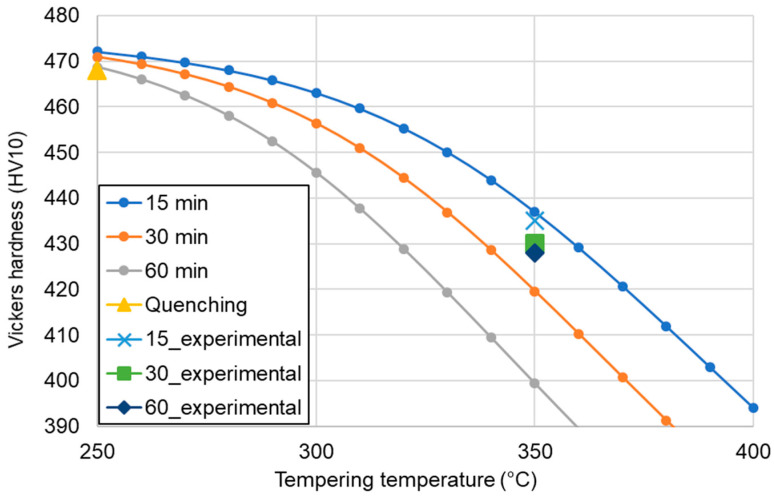
Comparison of the hardness results from the JMatPro simulations and the experiments.

According to the JMatPro simulations of the hardness changes during tempering for different times (Figure 9), the hardness should decrease with increasing time. Specifically, the hardness of the steel tempered at 350 °C for 15 min should be ~435 HV10, with a progressive decrease to 419 and 400 HV10 as the tempering time was increased to 30 and 60 min, respectively. The experimental results show that the hardness of the quenched martensitic microstructure was 468 HV10, which decreased to 428 HV after 60 min of tempering at 350 °C. This means that the value of the decrease was not significant. Regardless of the exact values, the tendencies of the calculation and experimental results match but not their magnitudes. While the calculations predicted a more pronounced drop in Vickers hardness as the tempering time increased, the experimental results showed that this drop was limited, revealing a high tempering resistance of the martensite in the 4% Mn steel studied in this work.

## 4. Conclusions

The dilatometric and microstructural analysis of the tempering process in Al-rich, medium-Mn steel was performed. The martensite in the analyzed 4% Mn steel had a high resistance to the tempering over 15, 30, and 60 min at 350 °C. An increase in the time from 15 to 60 min led to a higher contraction during tempering but this effect was neglectable. This means that a very small amount of cementite precipitated during tempering. According to the microstructure investigation, some interlath carbides were visible in the martensite laths after 60 min. A small thickening effect of the martensite laths with increasing tempering time was observed. The relatively small drop in hardness level by 40 HV after tempering for 60 min compared to the quenched state indicated the strong resistance of the 4% Mn steel to the tempering at 350 °C.

The results show that the martensite in the investigated medium-Mn steel was relatively thermally stable at tempering treatments up to 350 °C. It preserved good mechanical properties in this temperature region. This knowledge is important for designing novel heat treatment schedules, such as quenching and partitioning or austempering, especially for the heat treatments performed near the M_s_ temperature of medium-Mn steels.

## Figures and Tables

**Table 1:** Chemical composition (wt%) of the analyzed steel.

C	Mn	Si	Al	Mo	Nb
0.18	3.6	0.23	1.7	0.2	0.04
